# Vitamin D Effects on Osteoblastic Differentiation of Mesenchymal Stem Cells from Dental Tissues

**DOI:** 10.1155/2016/9150819

**Published:** 2016-11-13

**Authors:** Francesca Posa, Adriana Di Benedetto, Graziana Colaianni, Elisabetta A. Cavalcanti-Adam, Giacomina Brunetti, Chiara Porro, Teresa Trotta, Maria Grano, Giorgio Mori

**Affiliations:** ^1^Department of Clinical and Experimental Medicine, Medical School, University of Foggia, Foggia, Italy; ^2^Section of Human Anatomy and Histology, Department of Basic and Medical Sciences, Neurosciences and Sense Organs, University of Bari, Bari, Italy; ^3^Institute of Physical Chemistry, Department of Biophysical Chemistry, University of Heidelberg, Heidelberg, Germany; ^4^Max Planck Institute for Intelligent Systems, Stuttgart, Germany

## Abstract

1*α*,25-Dihydroxyvitamin D_3_ (1,25(OH)_2_D_3_), the active metabolite of vitamin D (Vit D), increases intestinal absorption of calcium and phosphate, maintaining a correct balance of bone remodeling. Vit D has an anabolic effect on the skeletal system and is key in promoting osteoblastic differentiation of human Mesenchymal Stem Cells (hMSCs) from bone marrow. MSCs can be also isolated from the immature form of the tooth, the dental bud: Dental Bud Stem Cells (DBSCs) are adult stem cells that can effectively undergo osteoblastic differentiation. In this work we investigated the effect of Vit D on DBSCs differentiation into osteoblasts. Our data demonstrate that DBSCs, cultured in an opportune osteogenic medium, differentiate into osteoblast-like cells; Vit D treatment stimulates their osteoblastic features, increasing the expression of typical markers of osteoblastogenesis like RUNX2 and Collagen I (Coll I) and, in a more important way, determining a higher production of mineralized matrix nodules.

## 1. Introduction

Vitamin D (Vit D) is crucial for many biological processes, that is, the bone mineralization of vertebrates, the maintenance of calcium homeostasis, cell proliferation, and differentiation.

The actions of Vit D are carried out by its active metabolite, 1,25-dihydroxycholecalciferol [1,25(OH)_2_D_3_ or calcitriol], that is produced through a series of enzymatic steps starting from cholecalciferol or vitamin D_3_.

The main portion of vitamin D_3_ is derived from the conversion of 7-dehydrocholesterol (Provitamin D) after the exposure of the skin to ultraviolet radiation. Age, skin surface exposed to the sun, thickness, and irradiation time are all factors that control Vit D synthesis [1].

Foods provide only few units of Vit D compared to the amount produced by the skin in response to sunlight.

Vit D is fat-soluble and is absorbed in the duodenum and jejunum and subsequently distributed through the lymphatic circulation [1].

1,25(OH)_2_D_3_ exerts its action by binding to a specific nuclear receptor, that is, the vitamin D receptor (VDR). It is a member of the class II steroid hormones [2].

Mice with targeted ablation of the nuclear VDR (conventional-VDR knockout mice) represent an important model to study the actions of the system 1,25(OH)_2_D_3_/VDR. The KO mice showed, after weaning, alopecia, an early development of hypocalcemia, which in turn induces a state of secondary hyperparathyroidism, infertility, and severely impaired bone formation: these are typical features recurrent in humans with vitamin D-dependent rickets type II [3–6].

Osteoblasts are among the cells expressing VDR; therefore they represent a functional target of 1,25(OH)_2_D_3_ action.

1,25(OH)_2_D_3_ can affect human osteoblast growth and differentiation stimulating bone formation and mineralization [7]. Moreover 1,25(OH)_2_D_3_ modulates bone deposition process preventing excessive and pathological mineralization [8].

The effects of 1,25(OH)_2_D_3_ on bone matrix protein expression have been also studied in cultures of calvaria cells, comparing young cells with more differentiated ones for different time periods. Following treatment with 1,25(OH)_2_D_3_, mature osteoblasts were more inhibited, favoring those which were in an earlier state of differentiation [9].

Vit D effects on osteoblast differentiation may be dissimilar according to the animal species considered; a discrepant responsiveness has been shown between two categories: human/rat osteoblasts and murine osteoblasts. Thus, in contrast to the stimulatory effect of Vit D on human and rat osteoblasts, Vit D has been demonstrated to determine an inhibitory effect on murine osteoblasts [10].

Vit D has been reported to induce alkaline phosphatase activity and to increase Collagen Type I expression in the course of proliferation and differentiation of human osteoblasts; this effect is less evident when lower concentrations are used [11].

Kveiborg et al. have shown that 1,25(OH)_2_D_3_ is able to offset, in aging osteoblasts* in vitro*, the reduction of gene expression which is normally required for osteoblast functionality [12].

Osteoblasts precursors are present on periosteal surface and in bone marrow; moreover they can differentiate from Mesenchymal Stem Cells (MSCs).

Adult stem cells are pluripotent cells capable of regenerating different tissues. Bone marrow contains cells with a high clonogenic capacity, the Colony-Forming Units Fibroblasts (CFU-Fs), which give rise to the cells of bone tissue, cartilage tissue, adipose, and fibrous tissues [13]. These CFU-Fs cells are called MSCs.

MSCs can be induced to differentiate into osteocytes, adipocytes, or chondrocytes [14] and also neuronal cells [15] or hepatocytes [16].

It is estimated that about 15% have a “stem” power, and among them there are those who can give origin to osteoblasts [13]. The commitment of MSCs towards differentiated cell lines is regulated by transcriptional mechanisms called master switches.

RUNX2 is a transcription factor with a key role in the control of osteoblast differentiation and function.

1,25(OH)_2_D_3_ is an important regulator of the RUNX2, with which it cooperates in inducing the expression of osteocalcin, that is, the key protein regulating bone matrix mineralization [17].

There are a lot of data in literature about the effect of Vit D on MSCs isolated from bone marrow, in particular concerning their osteogenic differentiation.

1,25(OH)_2_D_3_ influences human MSCs (hMSCs) inhibiting cell proliferation and stimulating their differentiation into osteoblasts [18, 19]. It has also been demonstrated, in hMSCs preimplantation cultures, that Vit D is able to determine the formation of mature osteoblasts, resulting in an increase of mineralized matrix formation, but after implantation of these cells the same Vit D is not sufficient in enhancing bone formation [20]. That leads to assuming a different* in vivo* effect of Vit D.

In order to overcome some negative aspects connected with the use of MSCs from bone marrow, that is, morbidity, pain, and low yield of cells, other sources of MSCs have been examined.

MSCs can be isolated from tissues different from the bone marrow such as adipose tissue, brain, skin, liver, and several fetal tissues; little is known about Vit D influence in osteogenic differentiation of MSCs from these alternative sources. Anyway, with MSCs from adipose tissue, the capacity of Vit D in osteoblast formation has been confirmed [21].

According to the evidence based research dental tissues represent an alternative and promising source of postnatal MSCs [22–26].

Deciduous teeth, or the wisdom tooth, that is a tooth with a limited chewing function and that often creates problems of overcrowding, can be used to isolate MSCs. In the case of immature teeth, a productive source of MSC is the dental bud (DB) that consists in noncalcified tissues; in the case of the mature tooth the sources are the periodontal ligament [27], the Dental Pulp (DP) [27–30], and the apical papilla [31].

Dental Pulp Stem Cells (DPSCs) are obtained from wisdom teeth pulp of adult donors, while Dental Bud Stem Cells (DBSCs) come from the DB. The DB, which is the immature form and therefore not yet fully calcified of the tooth, is an excellent source of stem cells; since it is considerably larger, it is comprised of cells more undifferentiated than the ones composing the pulp and can be removed in children of age between 8 and 12 in case of expected overcrowding with a safe technique called piezosurgery.

The advantage of the tooth bud, compared to the pulp, is first of all dimensional, it contains a greater number of cells, and moreover almost all the tissue is made from stem cells that have a high proliferative capacity and an excellent degree of stemness [24, 32, 33].

The tissues constituting the mature tooth, cement and periodontal ligament, enamel, dentin, pulp, and the central part of the bud, corresponding to the dental papilla, are all derived from DBSCs, obtained from the DB, thus containing MSCs which can successfully differentiate into osteoblast-like cells.

DBSCs are more undifferentiated MSCs, if compared to the ones isolated from the bone marrow; for this reason they can be considered an ideal model for studying the early stages of the osteoblastic differentiation process.

The differentiation into osteogenic lineage has already been demonstrated in DPSCs [27–29, 34] and a considerable mineral matrix deposition has been observed using innovative scaffolds for their culturing [35, 36]; moreover these cells, if cultured on *β*-tricalcium phosphate/poly (l-lactic acid/caprolactone) three-dimensional scaffolds, showed an increased osteogenesis in the presence of Vit D compared to dexamethasone [37].

The aim of this study is to analyze the effect of the active metabolite of Vit D, 1,25(OH)_2_D_3_, on DBSCs which represent a useful model to understand the anabolic activity of the bone cells, starting from very undifferentiated osteoblasts precursors.

## 2. Patients, Materials, and Methods

### 2.1. Materials

1*α*,25-Dihydroxyvitamin D_3_, ascorbic acid, *β*-glycerophosphate, dexamethasone, Alizarin Red powder, and alkaline phosphatase kit were from Sigma Aldrich, Milan, Italy.

Anti-RUNX2 antibody was from Abnova, anti-Coll I, anti-OPN, and anti-BSP II were from Abcam, Cambridge, UK.

### 2.2. Patients and Cell Cultures

Normal human third molar buds were collected from tooth buds of 10 healthy pediatric patients, 8–12 years of age, that underwent extractions for orthodontic reasons, mainly overcrowding, after informed consent from both patient parents.

The central part of DBs, corresponding to the dental papilla, was cut in small pieces and digested with agitation for 1 hour at 37°C in a solution of 3 mg/mL type I collagenase plus 4 mg/mL dispase (Gibco Ltd., Uxbridge, UK). Single-cell suspension was obtained by passing the cells through a 70 *μ*m BD Falcon strainer (Falcon) (Becton & Dickinson, Sunnyvale, CA).

After filtration, single-cell suspension was centrifuged at 1300 rpm for 5 min; the pellet was resuspended and cultured in Mesenchymal Stem Cell Culture medium supplemented with 5% fetal bovine serum (FBS), 100 U/mL penicillin-G, and 100 *μ*g/mL streptomycin (Gibco Limited, Uxbridge, UK). Cells were seeded at a density of 5 × 10^3^ cells/cm^2^.

Flasks were incubated at 37°C and 5% CO_2_ and the medium was changed every 3 days.

In order to induce osteoblastic differentiation, 1500 cells/cm^2^ were seeded and cultured in osteogenic medium consisting of *α*-MEM supplemented with 5% FBS, 10^−8 ^M dexamethasone, and 50 *μ*g/mL ascorbic acid (Sigma Aldrich, Milan, Italy).

For the evaluation of DBSCs ability to form mineralized matrix nodules* in vitro*, cells were cultured in the osteogenic medium supplemented with 10 mM *β*-glycerophosphate (Sigma Aldrich, Milan, Italy).

1,25(OH)_2_D_3_ (Sigma Aldrich, Milan, Italy) was reconstituted at 10^−4 ^M in 95% ethanol and stored at −20°C.

For all cell cultures, a 95% ethanol (vehicle) control was included at a concentration equivalent to that of Vit D. Thus cells were cultured in replicates with 1,25(OH)_2_D_3_ and an equivalent dilution of 95% ethanol.

### 2.3. Alizarin Red Staining (ARS)

The ability of DBSCs to generate mineralized matrix nodules* in vitro*, a feature normally attributed to osteoblasts, was assessed by performing Alizarin Red Staining on cells cultured for 21 days in osteogenic medium.

Thus, cells were gently rinsed with PBS and fixed with 10% formaldehyde at room temperature for 10 min.

Then they were washed twice with deionized water and incubated with 1% ARS solution for 10 min at room temperature. Cells were afterwards washed twice with deionized water, to remove excess staining, and air dried. The monolayer appeared to be red stained.

To quantify the ARS, cells were incubated with 10% acetic acid at room temperature for 30 min with shaking. The cell layer was scraped and vortexed and then the solution was incubated for 10 min at 85°C. After 5 min on wet ice, the suspension was centrifuged at 20,000 ×g for 15 min and 500 *μ*L of the supernatant was treated with 10% ammonium hydroxide to neutralize the acid. The Optical Density (OD) was read in triplicate at 405 nm.

### 2.4. Alkaline Phosphatase (ALP)

The expression of alkaline phosphatase, which is a marker of osteoblast differentiation, was assessed with the Leukocyte Alkaline Phosphatase kit (Sigma Aldrich).

Briefly, cell media were removed and cells were fixed in 0.5 mL of a fixative solution for 5 min at room temperature, according to manufacturer's instructions.

Subsequently, the wells were washed with deionized water and cells were stained with 0.3 mL ALP solution (a mixture of FRV-Alkaline Solution, Naphthol AS-BI Alkaline Solution, Sodium Nitrite Solution) in each well. Following a 15 min incubation in the dark, the wells were washed again with deionized water and air dried and cells were then inspected under the microscope.

### 2.5. Western Blot

Detection of osteoblastic markers as protein levels was performed by SDS-PAGE gel electrophoresis and western blot analysis. DBSCs were lysed after 7, 14, and 21 days of osteogenic differentiation; the cell lysates were cleared with a centrifugation at 13000 rpm for 15 min at 4°C. The total protein concentration of the supernatant was determined using a protein assay (BIORAD).

Equal amounts of protein for each sample were separated by SDS-PAGE and transferred to nitrocellulose membranes (Amersham, UK) with a Trans-Blot (Biorad, USA). The membranes were probed with primary antibodies overnight at 4°C and then samples were incubated for 90 min with secondary antibodies conjugated to horseradish peroxidase at room temperature. The reaction was analyzed with the Odyssey Infrared Imaging System of LI-COR (LI-COR Biotechnology Lincoln, Nebraska, USA).

### 2.6. Statistical Analyses

Statistical analyses were performed by Student's *t*-test with the Statistical Package for the Social Sciences (spssx/pc) software (SPSS, Chicago, IL, USA). The results were considered statistically significant for *P* < 0.05.

## 3. Results

### 3.1. ALP Positivity and Calcium-Rich Deposits in DBSCs

In order to investigate Vit D ability to induce the differentiation of DBSCs into osteoblasts, cells were cultured in osteogenic medium and stimulated with 1,25(OH)_2_D_3_ 10^−8 ^M. This concentration has been observed as the most efficacious among Vit D physiological concentrations analyzed in previous experiments.

DBSCs cultures were stopped at 7, 14, and 21 days after continuous Vit D treatment in differentiating conditions.

We used histochemical assay to evaluate the expression of alkaline phosphatase, a marker of osteoblastic differentiation, a key enzyme in the process of osteodeposition.

**Figure 1:**
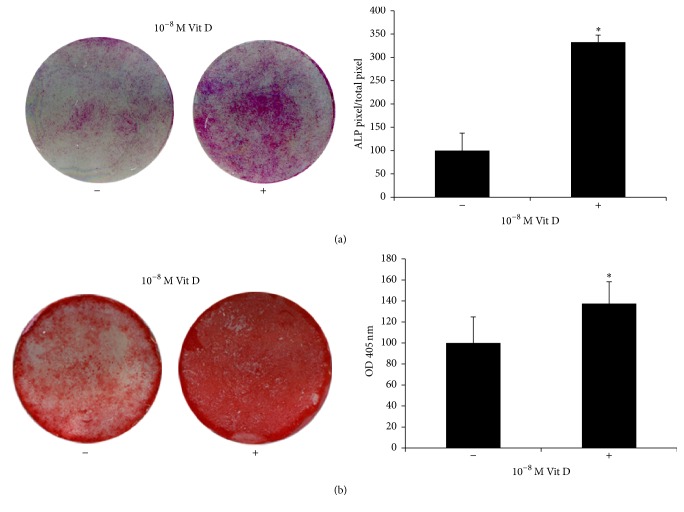
ALP positivity and mineralized nodules formation. (a) ALP histochemical assay (purple staining) performed on DBSCs seeded and differentiated for 7 days with vehicle (−) and 1,25(OH)_2_D_3_ (+). The graph shows quantification of positive staining as percentage compared to untreated cells (−) (^*∗*^ *P* < 0.05) and is representative for 3 independent donors. Data are presented as mean ± SEM. Student's *t*-test was used for single comparisons. (b) Alizarin Red (red staining) shows mineralized nodules formation by DBSCs incubated for 21 days in osteogenic medium with vehicle (−) and 1,25(OH)_2_D_3_ (+). The graph shows quantification of ARS with the Optical Density (OD) at 405 nm (b); ^*∗*^indicates statistical significance (*P* < 0.001). Data are representative for 3 independent donors and are presented as mean ± SEM. Student's *t*-test was used for single comparisons.

We found that DBSCs cultured in an osteogenic medium and treated with 1,25(OH)_2_D_3_ expressed higher levels of ALP after 7 days, compared to those of the control (Figure 1(a)). There was a considerable increase of ALP levels after 14 days, with slight differences between vehicle and treatment (data not shown).

Moreover, long-term cultures of DBSCs demonstrated the capacity to form calcium-rich deposits; this activity, representing the osteoblast final step matrix secretion, was assessed with ARS quantification.

Interestingly DBSCs deposition of mineral matrix nodules was significantly higher in cells cultured with Vit D, compared to the control (Figure 1(b)).

### 3.2. Osteoblast Markers Expression in DBSCs

The main osteoblastic markers, such as Collagen I (Coll I), its precursor Pro-Collagen I (Pro-Coll I), RUNX2, Bone Sialoprotein (BSP), and Osteopontin (OPN), were analyzed in DBSCs during the different steps of their osteogenic differentiation.

RUNX2 is the master gene of osteogenic differentiation; it directs MSCs to an osteoblastic lineage and inhibits their differentiation into other lineages such as adipocytes and chondrocytes [38].

**Figure 2:**
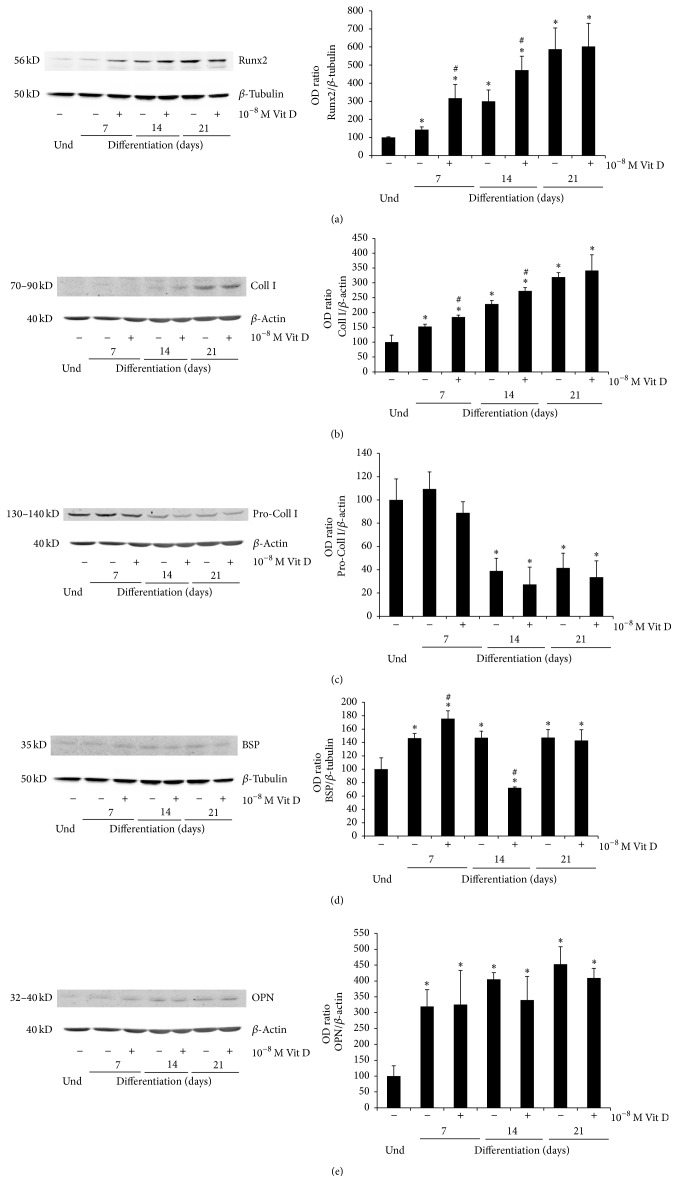
Protein expression. Immunoblots showing the trend expression of RUNX2 (a), Coll I (b), Pro-Coll I (c), BSP (d), and OPN (e) during the osteogenic differentiation process (0–21 days) of DBSCs cultured in osteogenic medium with vehicle (−) and 1,25(OH)_2_D_3_ (+). Each graph represents means ± SEM of 3 independent experiments. ^*∗*^ *P* < 0.01 compared to undifferentiated samples (und) and ^#^ *P* < 0.05 compared to untreated samples (−). Student's *t*-test was used for single comparison.

As shown in Figure 2(a), RUNX2 expression raised in a quite constant way during DBSCs osteogenic differentiation; however the addiction of Vit D greatly increased its expression level after 7 and 14 days of treatment. At 21 days of culture, no difference between vehicle and Vit D was observed.

A trend similar to the one already described for RUNX2 was observed for Coll I (Figure 2(b)): Vit D upregulated its expression in the first 7 days of differentiation; the effect was still present, although attenuated, after 14 and 21 days of treatment.

Interestingly Pro-Coll I, the immature precursor form of Coll I, showed a reverse trend (Figure 2(c)): Vit D reduced its expression at 7, 14, and 21 days of culture suggesting that this treatment promotes the conversion of Pro-Coll I in the mature Coll I.

It is remarkable how these data confirmed the typical trends of Coll I and its precursor during osteoblastogenesis, indicating that DBSCs are a reliable model of osteoblastic differentiation: moreover Vit D can sustain and increase this process.

Then we evaluated the expression of OPN and BSP which are considered the most important noncollagenous proteins produced by bone cells during the formation of bone matrix* in vitro* [39–41].

The expression of BSP was slightly upregulated after 7 days of Vit D treatment, while at 14 days there was a remarkable reduction; at 21 days BSP did not exhibit significant expression changes (Figure 2(d)).

No significant effect was attributed to the Vit D treatment on the expression of OPN (Figure 2(e)).

## 4. Discussion

The main actions of Vit D are those concerning mineral and skeletal homeostasis. The protracted deficiency of Vit D has different skeletal consequences in humans determining decreased bone mass and mineralization, with the manifestation of diseases known as Rickets in children and osteomalacia in adults [42, 43]. Low levels of Vit D also result in osteopenia or osteoporosis mainly attributed to an increased osteoclast bone resorption. These effects depend mostly on the indirect actions of 1,25(OH)_2_D_3_. Vit D may affect the bone indirectly, stimulating the intestinal calcium absorption, or directly, that is, by acting on bone cells; which of the two actions is prevailing is still debated [44] and the mechanisms whereby Vit D affects osteoblasts are mostly unknown [45].

Some studies have described 1,25(OH)_2_D_3_ as being able to increase human osteoblasts mineralization, leading to earlier and higher rate of mineral deposition [46], and able to accelerate hMSCs commitment with the subsequent osteoblast maturation [47, 48].

The bone marrow has been considered the major source of MSCs; however these cells can be obtained from other tissues and organs, which in some cases could represent an easier harvesting site being perceived as less invasive for donors. This is the case of stem cells isolated from the dental tissues.

Contrary to other sources used in adults to obtain MSCs, the dental tissues are formed at an older age, this is due to the late completion of odontogenesis and tooth eruption; consequently these tissues contain a higher amount of stem cells that have been discovered to be multipotent cells [22, 49]. DPSCs and DBSCs can be isolated from the third molar, in adult donors or in children, respectively. The dental bud is an immature organ, which originates several years after birth and is composed of undifferentiated cells with a higher proliferation rate than that observed for MSCs from bone marrow.

DBSCs fully satisfy the requirements to be considered MSCs; in fact it has been shown that they express more than 95% of mesenchymal stem markers (CD44, CD73, CD90, CD105, CD146, and HLA-I) and express the typical mesenchymal adhesion molecules [33]. Therefore all these mesenchymal features of DBSCs, together with their easy accessibility, make MSCs from dental tissues an excellent substitute to the bone marrow cells.

We have already demonstrated that osteoblastogenesis can successfully, and in a very productive way, take place from dental follicle, which is the peripheral part of the dental bud [32] and from dental bud [33]. These cells differentiate toward an osteoblastic phenotype; they express RUNX2, Coll I, and ALP, characteristic osteoblast markers, and produce mineralized matrix nodules.

In this work we analyzed how Vit D could influence the osteogenic differentiation of DBSCs. To this purpose we studied the expression of typical osteoblastic markers and mineral matrix deposition during DBSCs osteogenic differentiation in the presence, or not, of 1,25(OH)_2_D_3_. Our results confirmed the functional osteogenic differentiation of DBSCs [33]; in addition in this work we demonstrated that Vit D treatment enhances the commitment of DBSCs toward osteoblastic lineage. Indeed we observed that DBSCs treated with 1,25(OH)_2_D_3_ expressed increased levels of the main osteoblastic markers, RUNX2, Coll I, and ALP. Furthermore our results showed that enhanced commitment of DBSCs in presence of Vit D was also accompanied with an augmented production of mineralized matrix.

The enhanced expression of RUNX2, which is an early marker of osteoblastic differentiation, indicated that Vit D acts on uncommitted cells, prompting them to differentiate toward osteogenic lineage and then to express the typical osteoblastic markers ALP and Coll I. These proosteogenic effects exerted by 1,25(OH)_2_D_3_ on undifferentiated mesenchymal progenitor cells result subsequently in an accelerated formation of mineralized matrix nodules* in vitro* after 21 days of differentiation.

Our results also showed that the expression of RUNX2 and Coll I was accentuated in cells treated with Vit D during the early phases of osteogenic differentiation (7–14 days), while their expression turned to the control levels around 21 days of culture, indicating that the effects of Vit D are predominant at the beginning of the culture.

These results suggest that Vit D probably acts on the first steps of MSCs differentiation toward the osteoblastic phenotype, becoming less efficacious on differentiated cells. Our data are in line with previous data on human osteoblasts that the effect of Vit D depends on the time and the cells differentiation phase seeming to vanish in ongoing mineralization [46].

Our observations demonstrated that also stem cells from dental tissues respond to Vit D signal and are consistent with the data in literature which attribute to Vit D a key role in inducing osteogenic differentiation of MSCs [18, 47, 50] from human bone marrow. These results confirm that MSCs from dental tissues share similar features with MSCs from bone marrow and suggest that cultures of DBSCs in presence of Vit D could be taken in consideration for bone regenerative therapies.

Thus these data reflect the mesenchymal origin of DBSCs and their osteogenic capacity; moreover this study shows that osteoblastic differentiation of DBSCs was stimulated by 1,25(OH)_2_D_3_; our observations suggest that Vit D acts directly on these cells that can be considered osteoblast precursors, directing them to an increased bone matrix deposition.

The point of force of this work is that DBSCs are postnatal stem cells more undifferentiated than those isolated from bone marrow, that is, adult stem cells comparable to embryonic stem cells. The limit of our data is that all these conclusions have been already reported for MSCs from bone marrow; they are anyway a confirmation.

## 5. Conclusion

It is known that the efficacy of calcium intake on osteoporosis and fracture prevention is conditioned by the concomitant assumption of Vit D. Without adequate Vit D assumption only the 10–15% of the calcium can be utilized for building new bone [51].

Our finding that Vit D stimulates osteoblastic differentiation of DBSCs with the subsequent increase of bone mineral matrix deposition suggests in addition a possible use of Vit D as food aid in reconstructive therapies of bone with MSCs.

Widening the 1,25(OH)_2_D_3_ intake in the population could be recommend for both preventive and therapeutic purposes in bone diseases and trauma.

## Abbreviations

MSCs: Mesenchymal Stem Cells
DB: Dental bud
DBSCs: Dental Bud Stem Cells
Vit D, 1,25(OH)_2_D_3_: Vitamin D
VDR: Vitamin D receptor
Coll I: Collagen I
Pro-Coll I: Pro-Collagen I
BSP: Bone Sialoprotein
OPN: Osteopontin
DP: Dental Pulp
DPSCs: Dental Pulp Stem Cells
DFSCs: Dental Follicle Stem Cells
ALP: Alkaline phosphatase
ARS: Alizarin Red Staining.
